# CORESS Feedback: Cases from the Confidential Reporting System for Surgery

**DOI:** 10.1308/rcsann.2024.0112

**Published:** 2025-01-01

**Authors:** H Corbett

**Affiliations:** on behalf of the CORESS Advisory Board

## Abstract

CORESS is an independent charity, supported by AXA Health. We are grateful to those who have provided the material for these reports. The online reporting form is available via the CORESS website (coress.org.uk), which also includes previous Feedback reports. Published cases are acknowledged by a Certificate of Contribution, which may be included in the contributor’s record of continuing professional development, or which may form part of appraisal or annual review of competence progression portfolio documentation. Contributions from surgeons in training are particularly welcome.

## Air embolism in coronary artery bypass perfusion error

### Case 299

Cardiac surgery is a highly technical area in which a patient undergoing heart surgery may often be placed on heart–lung bypass to ensure continued organ oxygenation while the heart is temporarily stopped. In this case, a technical error gave rise to an adverse incident. On establishing cardiopulmonary bypass, the surgical team became aware that air had entered the heart after noting an air bubble in the cardioplegia cannula. The visible air was cleared and the operation continued. Postoperatively, it was evident that the patient had sustained a hypoxic brain injury. The cause was identified as a significant air embolism during institution of bypass. The patient did not regain consciousness after surgery. Following repeated computed tomography and clinical reviews, the prognosis was felt to be extremely poor. Treatment was withdrawn and the patient died.

The root causes were human error and equipment factors. While the specific clinical features of this case are not pertinent to all surgical practice, the principles of checking kit, ensuring knowledge of equipment operation prior to commencement of surgery and being aware of potential systems errors are relevant to all surgical practice. The Society of Clinical Perfusion Scientists (SCPS) is aware of and has commented on this case.

#### SCPS safety committee findings

This was an adverse event, arising in a highly technical and niche area of surgery, that involved misapplication of the perfusion apparatus. The equipment was checked and found to be functioning normally. Owing to the design of the pumps, the risk of human error leading to an inadvertent change of flow direction to the vent pipe was real. This potential to inadvertently reverse the flow on this particular pump was demonstrated to all members of the perfusion team.

It was recommended that once the heart–lung machine has been set up and existing safety checks have been completed, it should be switched to zero revolutions rather than placed in standby mode. This should be added to the perfusionist protocol checklist. Fluid should be aspirated into the vent line prior to its insertion (wet table test). This task should be included in normal checks undertaken by the scrub nurse. Human factors were contributory to this incident. As part of the massive air embolism protocol review, the SCPS recommended introduction of one-way valves to the vent suction line. Other specific recommended actions included a wet table test of all suckers as standard to avoid errors. It was felt that wet testing of vents prior to insertion would further increase the safety of these devices.

## Double trouble: vascular patient with two drug charts drawn up in error

### Case 300

A 76-year-old male vascular patient with atrial fibrillation and a femoropopliteal bypass graft was inadvertently prescribed both apixaban and enoxaparin together. Although the prescriptions were on different charts, it was an attempt to ‘bridge’ the patient on to a direct oral anticoagulant (DOAC).

#### Reporter’s comments

Pharmacy advice made it clear that concomitant prescribing of two anticoagulants is contraindicated unless it is for a patient being started on warfarin for venous thromboembolism, or for an established warfarinised patient with a subtherapeutic or unstable international normalised ratio. DOACs have a rapid onset of action (hours, not days). When switching from a parenteral anticoagulant to a DOAC, the first dose should be given when the next dose of low molecular weight heparin was due or on cessation of an intravenous unfractionated heparin infusion. The higher initiation doses for both apixaban and rivaroxaban are not ‘loading doses’. All DOACs have short half-lives and this is a larger dose to cover the higher period of risk, acutely after a venous thromboembolism.

#### CORESS comments

The CORESS advisory board commented that electronic prescribing might have averted this issue. It was also noted that the discipline of checking all drug charts in use remains an important function of the daily ward round.

## Inadvertent bladder injury at orchidopexy

### Case 301

An eight-year-old boy presented for bilateral second-stage orchidopexy for intra-abdominal testes. The first stage, a Fowler–Stephens procedure, in which the testicular vessels are divided to allow hypertrophy of the accessory supply via the vas deferens, had been carried out several years earlier. There was an unusually long period between the first and second stages owing to the COVID-19 pandemic. The parents were keen for both testes to be moved to the scrotum during the same operation to avoid further anaesthesia.

The procedure was approached laparoscopically. The primary port was inserted without complication, infraumbilically, using an open technique. Insertion of two secondary ports was hampered by limited space despite using standard insufflation pressure and flows. Both testes were seen in the abdomen. The left testis was associated with a large hernia sac and was managed through an open groin incision after a laparoscopic procedure on the right side. Visualisation was challenging owing to the limited space so the pressure for the pneumoperitoneum was increased to 15mmHg although the working space was still unusually small. The testis was mobilised, and then a Veress needle and ‘STEP’ port sheath were passed up from the scrotum into the peritoneal cavity under vision. The needle did not pass immediately into the peritoneal cavity and required some manipulation. An 11mm ‘STEP’ port was passed up the sheath, and the testis was grasped via the port and brought down into the scrotum.

Postoperatively, there were no immediate concerns but the family was anxious and the child was kept in overnight. He did not pass urine and was clearly peritonitic the next morning. Blood results and imaging were in keeping with a bladder leak, which was confirmed at exploratory surgery. The edge of the bladder had been injured by the Veress needle inserted via the scrotum and there was a clear urine leak. The patient made an uneventful recovery once the bladder had been repaired.

#### Reporter’s and CORESS comments

The pressure required for the pneumoperitoneum was higher than normal and might have pushed the bladder out more laterally than usual. The Veress needle was brought up medial to the medial umbilical ligament and did not pass smoothly, providing a clue about possible mis-passage that was not picked up at the time. Bladder injury is a recognised complication of this procedure. In future, the approach should be lateral rather than medial to the medial umbilical ligament. The bladder should be empty prior to the key manoeuvre although the role of catheterisation in young male patients prior to surgery is controversial and should be dealt with on a case-by-case basis.

## PEG feeding tube resistant to removal

### Case 302

A 49-year-old woman underwent a Roux-en-Y gastric bypass for obesity. This resulted in good weight loss but unfortunately, the patient later developed recurrent attacks of hyperinsulinaemic hypoglycaemia. Other methods to try to control the reactive hypoglycaemia were attempted without success and so bypass reversal was considered. The patient then had a 16F percutaneous endoscopic gastrostomy (PEG) feeding tube inserted laparoscopically. This helped to control the hypoglycaemia, enabling the surgeon to plan conversion to sleeve gastrectomy. However, in the interim, the patient was not able to tolerate the tube owing to pain and asked for its removal after three months.

**Figure 1:**
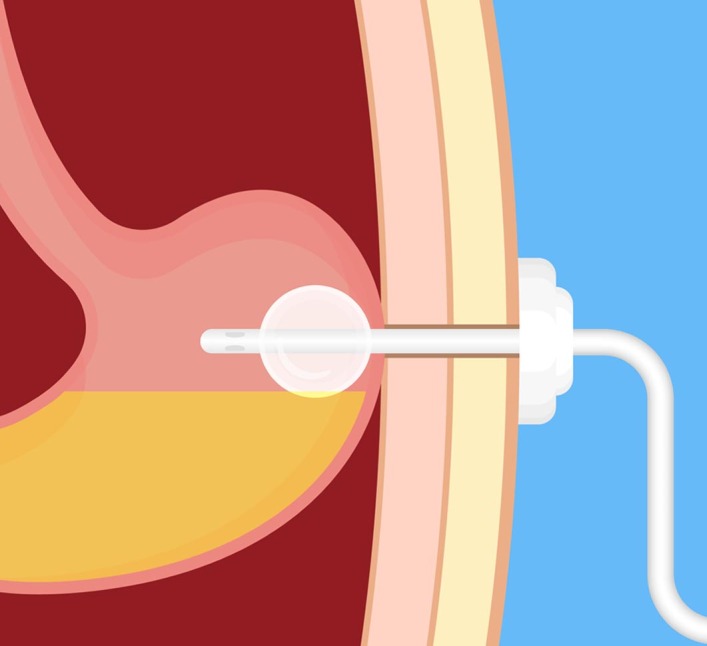
Before removing the PEG feeding tube, an internal balloon must be deflated. Image credit: Pepermpron/Shutterstock.com

The surgical registrar on call was tasked with removing the PEG tube, on a day-case list, under local anaesthesia. He attempted to do so, having checked that the tube rotated freely, but found the tube resistant to attempts to dislodge it. Rather than risk injuring the patient, he contacted the PEG team, who had not been involved with the placement of the tube. The PEG team explained that, with this particular device, it was necessary to deflate an internal balloon prior to device removal, a facet of the device with which the registrar was unfamiliar (Figure 1). The tube was subsequently removed with only minor discomfort to the patient. The case was discussed at a joint educational session between the PEG service and the surgical team involved.

#### Reporter’s comments

PEG tubes have various securing mechanisms preventing inadvertent dislodgement. In this hospital, the majority of PEG tubes are placed in situ by a dedicated PEG service. However, in this case, the tube had been placed by the upper gastrointestinal/bariatric team as a surgical procedure. The registrar who was asked to remove the tube was not provided with information necessary for the safe conduct of the procedure and had no access to information about the device employed. In the event, he made the sensible decision to desist from attempting removal against resistance and sought the advice of the PEG team.

#### CORESS comments

This case has several learning points. The registrar knew his limitations and made a sensible decision not to persist with tube removal when he was unfamiliar with the equipment and the procedure. The supervising consultant should not have delegated an unfamiliar procedure to a surgeon in training without either ensuring that he was confident to undertake this or providing adequate supervision. A record describing the type of PEG tube and implications for removal should have been included in the patient’s notes, facilitating eventual removal.

## Airway obstruction by haemostatic gauze

### Case 307

A patient was admitted to the cardiothoracic intensive care unit from their local hospital with retroperitoneal bleeding following anticoagulation for pulmonary emboli secondary to COVID-19 pneumonitis. In order to facilitate longer-term ventilation, the patient was taken to theatre for insertion of a tracheostomy. At the end of the procedure, absorbable haemostatic gauze was placed around the tracheostomy tube wound to stem skin-edge bleeding and the tube was secured. The patient returned to the cardiothoracic intensive care unit after the procedure.

Twenty-four hours later, the patient desaturated following routine repositioning and there were difficulties passing a suction catheter down the tracheostomy. An airway emergency was declared. The tracheostomy tube was removed. Attempts were made to ventilate the patient but these were unsuccessful and the patient lost output. Cardiopulmonary resuscitation was commenced. The patient was re-intubated with an endotracheal tube and return of spontaneous circulation was achieved. A bronchoscopy was then performed, revealing an obstruction at the base of the trachea. Saline flushes revealed a woven synthetic material (likely the absorbable haemostatic gauze), which had entered the airway during the resuscitation. Ventilation and oxygenation were compromised as a result. The material was successfully removed under anaesthesia and a further tracheostomy tube inserted.

#### Reporter’s and CORESS comments

Tracheostomy tubes are susceptible to complications including obstruction and displacement. Equipment facilitating tube replacement should always be kept at the bedside. Endoscopy to check tube siting and potential obstruction may help in planning further action. Various teams may be involved in tracheostomy tube siting and maintenance, including otolaryngology, maxillofacial surgery, anaesthetics and intensive care. Standardised procedures within an individual unit may reduce the risk of complications arising from error. Use of the haemostatic gauze employed in this case, around the tracheostomy, was not indicated and posed a potential risk of aspiration.

## Problems with drains

### Case 308

*Patient 1*: A 62-year-old man underwent elective sigmoid colectomy and defunctioning ileostomy. A corrugated drain was left in place in the left iliac fossa and brought out through the abdominal wall into a stoma bag. Two days after surgery, an entry in nursing notes recorded that the drain was shortened and cut, and that the stoma bag was removed because it was irritating the patient, who had mild dementia. There was no mention of drain removal. There was no mention in either the operation note or the nursing notes of use of a safety pin or suture to secure the drain and prevent retraction into the abdomen.

When assessed by the surgical team, the wound had been dressed and there was no sign of the drain. It was later noticed as an incidental finding on follow-up abdominal computed tomography that a piece of the drain had been retained in the left iliac fossa. The patient and relatives were informed, and the retained portion of drain was subsequently removed at re-look laparotomy.

*Patient 2*: A 72-year-old woman underwent laparotomy for colonic perforation due to diverticular disease. There was a sizeable pelvic collection and on completion of surgery, a tube drain was placed in the pelvis and secured to the skin with a suture by the surgeon. Nursing staff on the ward cut and bagged the drain. No safety pin was employed. On the ward round on the third postoperative day, the drain was noted to be absent. The retaining suture was still in the skin but had come undone. Radiography confirmed the drain to be lying loose in the peritoneal cavity. Further laparotomy was required to remove this.

*Patient 3*: A 26-year-old woman underwent appendicectomy and drainage of an appendix abscess. On completion of the operation, a Robinson tube drain was placed to the appendix bed and brought out though the abdominal wall. It was secured with a spiral suture wrapped around the drain and sutured to the skin next to the drain exit wound. The drain was left on free drainage and a stoma bag placed over it. On the second postoperative day, when the bag was changed, the drain was not visible although the silk ‘retaining’ suture was apparent, loosely coiled at its point of attachment to the skin. Drain removal from the abdominal cavity necessitated laparotomy.

#### Reporter’s comments

NHS England and NHS Improvement undertook a review of the National Reporting and Learning System in 2021, identifying nine reports of intra-abdominal wound drain shortening for closed drainage systems (‘cutting and bagging’) in which the drain had retracted into the abdomen. Suggested critical steps in the procedure, relevant to patient safety, include:

- Cut the drain approximately 7–10cm from the patient’s skin, allowing adequate protrusion.
- Place a sterile safety pin through the drain as close as possible to the skin (to prevent drain retraction) but not under tension against the skin, where it could cause an abrasion or pressure injury.
- Document in the patient’s notes that the drain has been cut and bagged. State clearly the drain length from skin exit site to drain end and the intra-abdominal site of drainage.
- On routine dressing change, observe the site for signs of drain retraction, withdrawal or infection. Report any unusual signs or complications and record in appropriate documentation.

#### CORESS comments

The CORESS advisory board agreed with the reporter’s comments. The operation note should clearly state where the drain is sited and should include a drain management plan, which should be communicated at handover to recovery staff and subsequently to ward staff. If multiple abdominal drains are used, each should be labelled.

## Wrong end of colostomy brought out

### Case 309

*Patient 1*: A 68-year-old man underwent laparoscopic defunctioning colostomy to treat a colovesical fistula. The stoma was inactive postoperatively but this was thought to be owing to a postoperative ileus. A suspicion was raised of a problem with the bowel on the morning of the fifth day after surgery and it was discovered later that day that the distal loop of bowel had been brought out as the stoma by a mistake, rather than the proximal end. The proximal bowel had been stapled and was therefore obstructed. The patient returned to theatre the same evening for laparotomy and refashioning of the stoma.

*Patient 2*: A 78-year-old woman presented unwell with a low sigmoid perforation secondary to diverticular disease. She had required three previous episodes of emergency surgery including formation of a loop colostomy, which had become ischaemic. This had been converted to an end colostomy before subsequently being successfully reversed.

On this occasion, the presumed distal end of the colon was stapled and the proximal end brought to the abdominal wall to form a stoma in an already scarred abdomen. After four days, the stoma had failed to produce any bowel content, and the patient had symptoms and signs of abdominal obstruction. Imaging demonstrated the technical error with what was in effect a mucous fistula at what had been thought to be the end colostomy site. The patient was taken back to theatre, the mucous fistula taken down and closed, and a new end colostomy fashioned from the proximal colon, which had been stapled. The patient required a prolonged stay on the intensive care unit.

*Patient 3*: The patient underwent surgery to form a defunctioning colostomy for an obstructing perforated carcinoma. The dissection was unexpectedly difficult owing to multiple adhesions, likely to have been secondary to a previous pelvic abscess. After 90 minutes of dissection, a loop of colon was brought up and divided, and the distal end stapled and dropped under the abdominal wall to facilitate a further definitive cancer operation after radiotherapy. However, the wrong end had inadvertently been stapled and the operating surgeon was unsure how the twist had happened. On day three, the patient had progressive abdominal distension and computed tomography demonstrated the error. The reporting surgeon felt that COVID-19 stresses may have had an impact as the theatre routines were severely disrupted.

#### CORESS comments

NHS England and NHS Improvement undertook a review of the National Reporting and Learning System between 2018 and 2021. Twenty relevant incidents were identified, detailing the distal rather than the proximal end of the bowel being used incorrectly to form the colostomy. Recognition of the problem occurred between two and six days after surgery, with a mode period of five days. Five cases were of documented laparoscopically performed procedures but no record of open versus laparoscopic surgery was available for the remaining reports. In 17 cases, return to theatre to correct the problem was necessary.

*Risk-reduction strategies*: Colorectal surgeons, nurses and surgical team members might consider the actions below to prevent or to identify and correct this technical error.

- Maintain vigilance when completing the finer technical steps involved in stoma creation, only delegating this task to junior or inexperienced members of the surgical team under proper supervision.
- Ensure that novice surgeons gain proficiency in end colostomy formation through supervised direct clinical experience, including during laparoscopic training.
- Mark the distal (or proximal) bowel limb intraoperatively using either a suture or cautery. Use the same method of marking the same bowel limb each time.
- Ask surgical team members to confirm identification of the proximal and distal bowel limbs whenever possible.
- Before closing the distal bowel limb, insert a red rubber or urinary catheter into the distal limb, infuse fluid and check to see whether the fluid drains from the patient’s anus.
- Towards the end of a laparoscopic procedure, reinsert the camera through the camera port, re-insufflate the abdomen and check to ensure that the proximal bowel limb is being pulled up to create the stoma.
- After closing the distal bowel limb, insert a flexible sigmoidoscope or colonoscope through the rectum to visualise the staple/suture line and confirm creation of a blind pouch.
- Once the stoma has been formed and opened at the end of the operation, instil water or air into the distal bowel limb through the rectum. If fluid or air is expressed through the stoma, the colostomy has been incorrectly formed using the distal bowel limb.
- Monitor the patient postoperatively to confirm the return of bowel sounds within 24–72 hours and the production of ostomy effluent within the first several days.
- Aside from absent or diminished bowel sounds and lack of ostomy effluent, assess the patient for additional signs and symptoms of bowel obstruction, including abdominal distension and pain.
- In patients with postoperative ileus lasting more than 36 hours, consider instilling a contrast enema through the stoma to identify errors in colostomy formation or other causes for obstruction.

